# Thirty Seventh Annual Convention, Detroit, September, 1900

**Published:** 1900-10

**Authors:** 


### A. V. M. A.

### THIRTY-SEVENTH ANNUAL CONVENTION, DETROIT, SEPTEMBER, 1900.

FOR many days prior to the opening session of the Thirty-seventh Annual Convention of the A. V. M. A. there assembled at Detroit veterinarians from all over the country. The time and occasion were fitting, and reached a section of our country that had not felt the influence of a meeting of the national body.

On the morning of September 4th when President Pearson called the convention to order he found on the sound of the gavel the largest number of veterinarians that had ever assembled in a veterinary convention in the United States.

The following list enumerates those who were in attendance at the several sessions of the convention:

Members: Drs. E. B. Ackerman, Brooklyn, N. Y.; A. H. Baker, Chicago, Ill.; Roscoe R. Bell, Brooklyn, N. Y.; George W. Bell, Kingston, Ont.; A. W. Bitting, Lafayette, Ind.; Eugene Burget, Wadsworth, Ohio; S. Brenton, Detroit, Mich.; Tait Butler, Indianapolis, Ind.; A. W. Clement, Baltimore, Md.; T. Bent. Cotton, Mt. Vernon, Ohio; R. R. Dinwiddie, Fayetteville, Ark.; W. H. Dodge, Leominster, Mass.; Wm. Dougherty, Baltimore, Md.; G. W. Dunphy, Quincy, Mich.; J. O. Greeson, Kokomo, Ind.; J. Hawkins, Detroit, Mich.; W. A. Heck, Maquoketa, Iowa; W. Horace Hoskins, Philadelphia, Pa.; R. S. Huidekoper, Washington, D. C.; J. W. Jamieson, Paris, Ky.; G. A. Johnson, Sioux City, Iowa; Wm. Jopling, Owosso, Mich.; W. H.

Kelly, Albany, N. Y.; M. E. Knowles, Helena, Mont.; G. Ed. Leech, Milwaukee, Wis.; Wm. Herbert Lowe, Paterson, N. J., W. J. Martin, Kankakee, Ill.; Frank C. McCurdy, St. Joseph; Mo.; J. C. McNeil, Pittsburg, Pa.; J. R. Mitchell, Evansville, Ind.; R. C. Moore, Kansas City, Mo.; E. M. Nighbert, Mt. Sterling, Ill.; J. D. Nighbert, Pittsfield, Ill.; Leonard Pearson, Philadelphia, Pa.; Austin Peters, Boston, Mass.; J. M. Phillips, St. Louis, Mo.; E. C. Porter, New Castle, Pa.; E. M. Ranck, Philadelphia, Pa.; L. N. Reefer, Wheeling, W. Va.; John J. Repp, Ames, Iowa; M. H. Reynolds, St. Anthony Park, Minn.; Wm. H. Richards, Emporia, Kansas; J. L. Robertson, New York City, N. Y.; James Robertson, Chicago, Ill.; D. E. Salmon, Washington, D. C.; V. Schaefer, Tekamah, Neb.; Walter Shaw, Dayton, Ohio; Edgar H. Shepard, Cleveland, Ohio; R. V. Smith, Frederick City, Md.; S. Stewart, Kansas City, Mo.; N. I. Stringer, Watseka, Ill.; W. J. Tomlinson, Williamsport, Pa.; George R. White, Nashville, Tenn.; W. L. Williams, Ithaca, N. Y.; J. F. Winchester, Lawrence, Mass.; A. Youngberg, Lake Park, Minn.

Visitors: Drs. Francis Abele, Quincy, Mass.; F. E. Anderson, Findlay, Ohio; W. J. Armour, Goshen, Ind.; G. W. Backner, Bluffton, Ind.; H. B. Baker, Detroit, Mich.; L. F. Baldcock, Birmingham, Mich.; H. B. Bascer, Lansing, Mich.; U. S. G. Bieber, Kutztown, Pa.; E. C. Beckett, Boston, Mass.; H. L. Bellinger, Plainwell, Mich.; Judson Black, Richmond, Mich.; F. M. Blatchford, Brighton, Mich.; J. H. Blattenberg, Lima, Ohio; W. S. Birden, Kingston, Pa.; S. E. Boulter, Niagara Falls, Ont.; J. W. Brodie, Pontiac, Mich.; L. A. Brown, Aylmer, Ont.; C. E. Brownback, Peru, Ind.; J. C. Burneson, Wooster, Ohio; E. H. Callander, Zanesville, Ohio; J. C. Callander, Parkersburg, W. Va.; A. Campbell, Jackson, Mich.; H. T. Carpenter, Ada, Ohio; M. R. Carr, Kirkton, Ont.; G. H. Carter, Saginaw, Mich.; U. S. Cass, St. Louis, Mo.; S. R. Craver, Youngstown, Ohio; J. B. Caughey, Columbus, Ohio; H. P. Clute, Marinette, Wis.; W. E. Clemons, Greenville, Ohio; L. L. Conkey, Grand Rapids, Mich.; A. S. Cooley, Cleveland, Ohio; A. Cornell, Elkton, Mich.; R. A. Craig, Lafayette, Ind.; D. Cummings, Port Huron, Mich.; D. W. Curtis, Detroit, Mich.; Albert E. Cunningham, Cleveland, Ohio; D. A. Davidson, Princeton, Ind.; C. F. Dawson, Detroit, Mich.; J. F. Deadman, Sault Ste Marie, Mich.; J. A. Dell, Ann Arbor, Mich.; W. F. Derr, Wooster, Ohio; D. S. DeWolf, Hart, Mich.; B. A. Dillahunt, Springfield, Ohio; J. Drury, Ypsilanti, Mich.; Mr. W. W. Dixon, New York City, N. Y.; Drs. Charles E. Edwards, St. Thomas, Ont.; J. T. Elliott, Detroit, Mich.;

Wm. Elliott, Hickory Corner, Mich.; Mr. Alex. Eger, Chicago, Ill.; Drs. E. W. Emery, Greenfield, Ohio; H. Emery, Pittsburg, Pa.; J. D. Fair, Berlin, Ohio; W. C. Fair, Cleveland, Ohio; Thos. Farmer, Grand Blanc, Mich.; S. N. Fitch, Auburn, Ind.; J. E. Foster, Coshocton, Ohio; E. T. Frank, Warren, Minn.; B. M. Freed, Sharon, Pa.; Charles B. Frederick, Columbus, Ohio; George Fitchett, Pinnebog, Mich.; L. Frothingham, Boston, Mass.; H. Fulstow, Norwalk, Ohio; L. D. Gibson, Adrian, Mich.; W. A. Giffin, Detroit, Mich.; F. G. Gilbank, Detroit, Mich.; H. M. Gillian, Mason City, Iowa; H. M. Gohn, St. Johns, Mich.; W. R. Granger, Plymouth, Mich.; J. S. Grant, Portland, Mich.; John W. Graves, Hamilton, Ont.; L. K. Green, Detroit, Mich.; L. A. Greiner, Indianapolis, Ind.; W. H. Gribble, Washington Court House, Ohio; W. S. Hamilton, Chelsea, Mich.; A. H. Hartwig, Watertown, Wis.; C. S. Hess, Wabash, Ind.; W. F. Heyde, St. Louis, Mo.; R. C. Hill, West Alexandria, Ohio; W. C. Holden, Delphos, Ohio; John Hooker, New Baltimore, Mich.; C. H. Howard, Calumet, Mich.; R. E. Hunt, Alma, Mich.; Thomas E. Jones, Newark, Ohio; James J. Joy, Detroit, Mich.; W. H. Kerr, Mansfield, Ohio; R. H. Kestell, Ypsilanti, Mich.; J. W. Klotz, Nobleville, Ind.; W. E. Krenler, Wadsworth, Ohio; T. G. Kuff, St. Louis, Mich.; J. Stewart Lacock, Allegheny, Pa.; R. W. McCally, New York City, N. Y.; M. C. McClain, Jeromeville, Ohio; F. McCoy, Chicago, Ill.; R. W. McDonald, Flint, Mich; Wm. McEachran, Windsor, Ont.; Alex. McGregor, Rockport, Ohio; A. McKercher, Lansing, Mich.; W. O. McHugh, Toledo, Ohio; W. R. McMurty, Janesville, Mich.; Wallace McQueen, Oxford, Mich.; E. D. McQueen, Lowell, Mich.; F. W. Menhennett, Ishpeming, Mich.; W. A. Meredith, Corry, Pa.; Thomas Meredith, Jamestown, N. Y.; A. E. Metzger, Clyde, Ohio; George C. Moody, Mason, Mich.; R. J. Morrison, Detroit, Mich.; C. A. Miller, Louisville, Ky.; S. A. Myers, Wilmington, Ohio; William F. Myers, Fort Wayne, Ind.; J. T. Nattress, Delavan, Ill.; M. C. Newburg, Hanover, Mich.; John V. Newton, Toledo, Ohio; Charles N. Nye, Coopersville, Mich.; C. L. Osgood, Samaria, Mich.; H. F. Palmer, Detroit, Mich.; A. J. Payne, Cincinnati, Ohio; S. A. Pinte, Lexington, Ky.; N. Rectenwald, Pittsburg, Pa.; A. Roberts, Waukesha, Wis.; J. E. Rodger, Anderson, Ind.; William Rose, Grand Rapids, Mich.; Frederick P. Ruhl, Fairmont, W. Va.; Wm. A. Rushworth, Chicago, Ill.; A. J. Savage, Colorado Springs, Col.; Chas. Schmitt, Dodgeville, Wis.; W. M. Simpson, Malden, Mass.; C. Rowland Simpson, Somerville, Mass.; C. S. Shier, Detroit, Mich.; D. King Smith, Toronto, Ont.; H.

S. Smith, Albion, Mich.; R. N. Steele, Chicago, Ill.; S. S. Snyder, Cedarburg, Wis.; C. N. Stowe, Saginaw, Mich.; C. C. Stevens, Yale, Mich.; A. G. Tegg, Rochester, N. Y.; A. H. Tanner, Ashtabula, Ohio; J. H. Tennent, London, Ont.; Thos. Thacke, Renfrew, Ont.; H. E. Titus, Lafayette, Ind.; F. Torrance, Winnipeg, Manitoba; W. J. Torrance, Cleveland, Ohio; W. H. Turner, North Amherst, Ohio; S. S. Treadwell, Englewood, N. J.; V. C. Vaughan, Ann Arbor, Mich.; Z. Veldhius, Tremont, Mich.; John Vhay, Detroit, Mich.; George Waddle, Hastings, Mich.; C. A. Waldron, Tecumseh, Mich.; H. C. Wann, Charlevoix, Mich.; J. E. Ward, Perry, Mich.; Mr. L. D. Watkins, Manchester, Mich.; Drs. George A. Waterman, Lansing, Mich.; F. C. Wells, Warren, Mich.; R. W. Whitehead, Youngstown, Ohio; A. W. Wier, Greensville, Pa.; Wm. M. Wilson, Hartstown, Pa.; W. J. Williams, Franklin, Ind.; W. E. Wight, Allegheny, Pa.; J. C. Whitney, Hillsdale, Mich.; P. W. Wooly, Lapeer, Mich.; I. A. Wynn, Kenton, Mich.; D. P. Yonkerman, Kalamazoo, Mich.; Mr. A. H. Zenner, Detroit, Mich.

Lady Visitors: Mrs. E. B. Ackerman, Brooklyn, N.Y.; Mrs. L. Brenton, Miss Lillian Brenton, Detroit, Mich.; Mrs. A. H. Baker, Chicago, Ill.; Mrs. G.W. Bell, Kingston, Ont.; Mrs. W. S. Brown, Pa.; Mrs. J. C. Burneson, Wooster, Ohio; Mrs. A. Campbell, Jackson, Mich.; Mrs. H. T. Carpenter, Ada, Ohio; Mrs. Cheesbrough, Detroit, Mich.; Mrs. F. A. Cooley, Cleveland, Ohio; Mrs. Cowing, Detroit, Mich.; Mrs. D. W. Curtiss, Cadillac, Mich.; Mrs. D. A. Davison, Princeton, Ind.; Mrs. J. A. Dell, Ann Arbor, Mich.; Mrs. W. F. Derr, Wooster, Ohio; Mrs. G. W. Dunphy, Quincy, Mich.; Mrs. H. Emery, Pittsburg, Pa.; Mrs. J. D. Fair, Berlin, Ohio; Mrs. W. A. Giffen, Detroit, Mich.; Miss Ella Gribble, Washington Court House, Ohio; Mrs. Guerney, Detroit, Mich.; Mrs. A. H. Hartwig, Watertown, Wis.; Mrs. W. A. Heck, Maquoketa, Iowa; Mrs. T. B. Hillock, Columbus, Ohio; Mrs. W. Horace Hoskins, Philadelphia, Pa.; Mrs. G. A. Johnston, Sioux City, Iowa; Mrs. W. H. Kelly, Albany, N. Y.; Mrs. G. Ed. Leech, Milwaukee, Wis.; Mrs. E. D. McQueen, Lowell, Mich.; Mrs. Wm. McEachran, Windsor, Ont.; Miss J. Meredith, Jamestown, N. Y.; Mrs. A. E. Metzger, Clyde, Ohio; Mrs. J. R. Mitchell, Evansville, Ind.; Mrs. A. Roberts, Waukesha, Wis.; Mrs. F. P. Ruhl, Fairmont, W. Va.; Mrs. E. H. Shepard, Cleveland, Ohio; Mrs. C. R. Simpson, Summerville, Mass.; Mrs. S. Stewart, Kansas City, Mo.; Mrs. J. H. Tennent, London, Ont.; Mrs. W. H. Turner, North Amherst, Ohio; Mrs.

R. A. Walster, West Eaton, Mass.; Mrs. L. D. Watkin, Manchester, Mich.; Mrs. J. C. Whitney, Hillsdale, Mich.; Mrs. W. E. Wight, Allegheny, Pa.; Mrs. A. W. Weir, Greenville, Pa.; Mrs. W. J. Williams, Franklin, Ind.; Mrs. John Vhay, Detroit, Mich

President Pearson found much pleasure in the privilege of introducing to the members assembled Mayor William C. Mayberry, of the city of Detroit. In welcoming the delegates and members of the profession to Detroit he cited the early history of the settlement and growth of the city, and paid a glowing tribute to the pioneers of that territory, touching upon the growth and progress of medicine and its broad character of to-day compared with the narrow attitude of those engaged in scientific medicine in the early years of its history, and most fittingly commended the generous, catholic-spirited disposition of those engaged in the practice of medicine to-day in conferring knowledge gained that all may profit by the same, the human race being the special beneficiary.

He commended the spirit and sentiment of those who gave much time and consideration to the welfare of the dumb animals in protecting them from cruelty, and the growth of this work through the great director, Henry Bergh, whose devotion to the humane aspect of this field of work had contributed more than history can ever recount, associated as his work was with that of the veterinary profession. He seemed well informed on the progress of veterinary medicine, and gave a most generous welcome to the members of the convention on behalf of the city, concluding with a wish that they would return home well pleased and well repaid with their deliberations in Detroit.

President Pearson then asked Dr. W. Horace Hoskins, of Philadelphia, to respond to the Mayor's cordial greetings. After an amusing reference to Mayor Mayberry's success in obtaining the mayoralty of Detroit and his own unsuccessful effort to obtain the same high honor in Philadelphia, Dr. Hoskins centred his remarks upon the grave responsibility that rests upon those who hold high official place in city, State, and nation in these times, when the growth of sanitary science points so clearly to one true plan and means of promoting and maintaining the health of the people, thus assuring a security and freedom from many dangers in an unguarded food-supply, situations that no longer hide from the scientific eye the insidious dangers and which brought with this era of advancement, ways and means of controlling and preventing the dangers resulting therefrom.

These introductory remarks were followed by the presentation of the President's address.[1]

In his review of the progress in general, the growth of the Association, and the many aspects of its work, he laid much stress upon the broader field of employmentof the veterinarian that might be made, and referred to veterinarians as *animal engineers* in the field of animal husbandry. The address was replete with excel lent thoughts, suggestions, and comments, and should be read and thoroughly considered by every veterinarian in the land.

The minutes of the preceding meeting at New York having been printed and placed at the command of every member, it was deemed unnecessary to read them at this meeting, and the President at once proceeded to call for a report of the Executive Committee. The following recommendations of the Executive Committee were received and approved by the Association:

First, the creating of two additional vice-presidents, making the number five instead of three.

The committee having decided the charges of the violation of the Code of Ethics in unprofessional advertising by Dr. W. E. Wight, of Allegheny, Pa., well founded, advised his expulsion, which was unanimously agreed to by the Association.

Charges of plagiarism made against Dr. George B. Blackman, of Georgia, were received. The Secretary was directed to instruct Dr. Blackman to appear at the next annual meeting to answer for the same.

In the charges against Dr. A. J. Kline, for advertising contrary to the Code of Ethics, action was suspended upon the request of Dr. Kline and assurance of freedom from the same in the future.

At a previous meeting of the Association the resignation of Dr. R. R. Dinwiddie was held over, with a request that the officers would urge his continuation as a member of the Association, which was favorably acquiesced in by Dr. Dinwiddie.

The resignation of Dr. Charles W. Heitzman, of Louisiana, he having engaged in the practice of human medicine, was, upon recommendation, accepted by the Association.

The proposal of Prof. Theodore Kitt for honorary membership was approved by the Executive Board, and under a suspension of the by-laws he was unanimously elected.

During the several meetings of the Executive Committee the following applications for membership were examined and favor-

[1] See page 521, September number.

ably recommended and the applicants elected to membership by the Association:

Francis Abele, Jr., V.S. (Ontario V. C., 1900), Quincy, Mass.; vouchers, E. C. Porter, James Robertson. F. E. Anderson, V.S. (Ontario V. C., 1886), Findlay, Ohio; voucher, Tait Butler. E. C. Beckett, M.D.V. (Harvard, 1887), Boston, Mass.; vouchers, A. Peters, J. F. Winchester, T. B. Cotton. J. H. Blattenberg, V.S. (Ontario V. C., 1892), Lima, Ohio; voucher, T. B. Cotton. J. C. Burneson, V.S. (Ontario V.C., 1891), Wooster, Ohio; vouchers, L. C. Porter, J. J. Repp, T. B. Cotton. William J. Butler, V.S. Ontario (V. C., 1889), Fond du Lac, Wis.; vouchers, G. W. Butler, R. H. Harrison. U. S. Cass, V.S. (Ontario V. C., 1892), St. Louis, Mo.; vouchers — —. Charles Caster, V.M.D. (University of Pennsylvania, Vet. Dept., 1894), E. Las Vegas, N. M.; vouchers, Tait Butler, S. G. Hendren. W. G. Clark, M.D.C. (Chicago V. C., 1893), Marinette, Wis.; voucher, R. H. Harrison. H. P. Clute, V.S. (Ontario V. C., 1887), Marinette, Wis.; voucher, R. H. Harrison. A. S. Cooley, D.V.S. (Chicago V. C., 1887), Cleveland, Ohio; vouchers, R. C. Moore, F. C. McCurdy, A. H. Baker. Robert A. Craig, D.V.M. (Iowa State College, 1897), Lafayette, Ind.; vouchers, A. W. Bitting, Tait Butler. E. C. Crevier, D.V.S. (Montreal V. C., 1883), Detroit, Mich.; voucher, S. Brenton. A. E. Cunningham, V.M.D. (University of Pennsylvania Vet. Dept., 1898), Cleveland, Ohio; voucher, E. M. Ranck. D. W. Curtis, V.S. (Ontario V. C., 1892), Cadillac, Mich.; voucher, G. W. Dunphy. D. A. Davison, D.V.S. (Chicago V. C., 1892), Princeton, Ind.; voucher, J. R. Mitchell. W. P. Ellenberger, D.V.S. (Columbian University, 1897), Wadsworth, Ohio; vouchers, G. R. White, W. C. Rayen. H. James Elliott, M.D.V. (McKillip V. C., 1900), Winnipeg, Manitoba; voucher, W. J. Hinman. William C. Fair, V.S (Ontario V. C., 1871), Cleveland, Ohio; vouchers, Walter Shaw, E. H. Shepard. Langdon Frothingham, M.D.V. (Harvard V. C., 1889), Boston, Mass.; vouchers, Austin Peters, J. F. Winchester. Harry Fulstow, V.S. (Ontario V. C., 1892), Norwalk, Conn.; voucher, Joseph Roberts. Johnson Gibbons, M.R.C.V.S. (Dick, 1880), Vancouver, B. C.; voucher, Charles H. Higgins. H. M. Gohn, V.S. (Ontario V. C., 1893), St. Johns, Mich.; voucher, George W. Dunphy. L. Kenneth Green, V.M.D. (University of Pennsylvania, 1894), Detroit, Mich.; vouchers, G. W. Dunphy, S. Brenton. John W. Groves, D.V.S. (McGill University, 1899), Hamilton, Ont.; voucher, Chas. H.

Higgins. Robert Hamilton, M.R.C.V.S. (Glasgow, 1888), Victoria, B. C.; voucher, Chas. H. Higgins. Joseph T. Hernsheim, V.M.D. (University of Pennsylvania Vet. Dept., 1897), Kenosha, Wis.; voucher, R. H. Harrison. R. W. Hewett, D.V.S. (A. V. C., 1894), Milwaukee, Wis.; voucher, R. H. Harrison. William F. Heyde, V.S. (Ontario V. C., 1894), St. Louis, Mo.; vouchers, E. M. Nighbert, J. Hawkins. C. H. Howard, M.D.V. (McKillip V. C., 1899), Calumet, Mich.; vouchers, G. W. Bell, G. W. Dunphy. Louis A. Klein, V.M.D. (University of Pennsylvania Vet. Dept., 1897), Philadelphia, Pa.; vouchers, Leonard Pearson, C. J. Marshall. J. Stewart Lacock, V.M.D. (University of Pennsylvania Vet. Dept., 1895), Allegheny, Pa.; vouchers, W. Horace Hoskins, Leonard Pearson. J. A. McCrank, D.V.S. (McGill University, 1891), Plattsburg, N. Y.; voucher, W. H. Kelly. Stewart W. McClure, V.M.D. (University of Pennsylvania Vet. Dept., 1898), Helena, Montana; voucher, W. Henry Kelly. William McEachran, D.V.S. (McGill University, 1880), Windsor, Ont.; vouchers, A. W. Clement, R. S. Huidekoper. C. M. McFarland, D.V.S. (Kansas City V. C., 1900), Cincinnati, Ohio; vouchers, R. C. Moore, S. Stewart. C. A. Miller, D.V.S. (Chicago V. C., 1890), Louisville, Ky.; vouchers, E. M. Nighbert, J. Hawkins. Robert J. Morrison, V.S. (Ontario V. C., 1892), Detroit, Mich.; voucher, S. Brenton. William F. Myers, D.V.S. (Chicago V. C., 1889), Fort Wayne, Ind.; voucher, J. R. Mitchell. G. E. Nesom, D.V.M. (Iowa State College, 1898), Clemson College, S. C.; voucher, Benjamin McInnes. L. A. Nolan, V.M.D. (University of Pennsylvania Vet. Dept., 1900), Baltimore, Md.; voucher, Wm. Dougherty. Frederick Ruhl, D.V.S. (A. V. C., 1895), Fairmont, W. Va.; vouchers, W. Horace Hoskins, Leonard Pearson. William H. Rushworth, M.D.C. (Chicago V. C., 1893), Chicago, Ill.; vouchers, Joseph Hughes, T. A. Bown. Charles Schmitt, M.D.C. (Chicago V. C., 1892), Dodgeville, Wis.; voucher, R. H. Harrison. H. C. Simpson, D.V.S. (Kansas City V. C., 1898), Denison, Iowa; voucher, S. Stewart. S. S. Snyder, D.V.S. (Chicago V. C., 1887), Cedarburg, Wis.; voucher, R. H. Harrison. Adolf Stauffer, V.S., Covington, Ky.; voucher, J. W. Jamison. James J. Summerfield, D.V.S. (University of California, 1897), Santa Rosa, Cal.; voucher, Fred. E. Peirce. Harry E. Titus, D.V.M. (Iowa State College, 1898), Lafayette, Ind.; vouchers, A. W. Bitting, Tait Butler. F. Torrance, D.V.S. (McGill University, 1882), Winnipeg, Manitoba; vouchers, A. W. Clement, R. S. Huidekoper.

L. Van Es, M.D., V.S. (Ontario V. C., 1893), Mobile, Ala.; voucher, C. A. Cary. A. R. Wake, D.V.M. (Iowa State College, 1894), Cudahy, Wis.; voucher, R. H. Harrison. D. P. Yonkerman, V.S. (Ontario V. C., 1881), Kalamazoo, Mich.; vouchers, Wm. Jopling, G. W. Dunphy.

Several applications were laid over pending further investigation of the qualifications of the applicants.

The report of the Executive Board was followed up by the committee reports. The first one responded to was that of Publications, through the Chairman, Dr. W. L. Williams, showing the cost of reproducing the proceedings of the Association and distribution of the copies printed.

The Committee on Army Legislation, through its Chairman, Dr. D. E. Salmon, presented its report.[1] After reviewing the vast work done by the committee the report closed with a most thoughtful and touching appeal to the members of the profession to continue in the good work, that its achievements may triumph at the next session of Congress.

(To be continued.)

---

Dr. G. W. Dunphy, of Quincy, Mich., seemed to be at every point during the convention, and responded to every demand and need of the visitors on that occasion.

Every Michigan veterinarian seemed to be loaded with ammunition and determined to spend its force on the JOURNAL.

That veteran Association member, Dr. Wm. Dougherty, of Baltimore, Md., became as spry as a football player under the soothing influences of the springs at Mt. Clemens, near the Convention city.

Dr. A. H. Baker, of Chicago, always stands up for the West and leads all the movements "agin" the East.

The serious, thoughtful countenance of Dinwiddie, of Arkansas, never portrays what is coming next when in an argumentative mood on the floor.

Dr. G. R. White, of Nashville, says that the new veterinary college of that city will not down for want of a faculty.

Dr. G. Ed. Leech, of Milwaukee, Wis., was the leader of the McKinley forces in that State.

Dr. T. Bent Cotton, of Mt. Vernon, Ohio, was often found in the hotel corridors, reclining on an easy couch, swapping horse stories with his colleagues of the Convention.

[1] Will be published in November number.

Dr. S. Brenton, of Detroit, Mich., was never happier than when enjoying the privilege of entertaining his colleagues at Detroit.

Dr. J. W. Jamieson, of Paris, Ky., was the sole representative of the "Blue Grass State," and found many willing listeners to the almost fairy-like tales of the thoroughbreds of "Old Kentucky."

Dr. D. E. Salmon proved a happy toast-master, and his versatile knowledge of his fellow-members made many telling points as he called them to respond on topics of professional interest.

An Atlantic City boom kept Lowe, of New Jersey, preoccupied and hustling all the time.

Drs. L. N. Reefer, of Wheeling, W. Va., and J. C. McNeil, of Pittsburg, Pa., were looked upon as well versed in political methods and plans.

Dr. J. F. Winchester, of Lawrence, Mass., met a fraternal companion at every point and place; he carried with him from Massachusetts a message of peace among the profession in the "Bay State."

Dr. John J. Repp, of Ames, Iowa, was hourly concerned as to the public attitude of the Convention on the leading questions of the profession as they exist in these days of rapid changes.

That hustling Montana man, Dr. M. E. Knowles, of Helena, says the Association must see the Yellowstone Park in 1902.

Strenuous Peters, of Boston, Mass., seemed weary of the quietude and peace that prevail over veterinary sanitary police measures in Massachusetts.

Dr. G. A Johnson, of Sioux City, Iowa, was hourly concerned over the rushing of the programme through on time.

Reynolds, of the Publication Committee, found his hands full in keeping track of the Convention work, directing the strength of the Western members of the profession, with his weather eye open for new points in control work.

Kelly, of Albany, N. Y., seemed lost without his old companion, Hinkley, of Buffalo, now of Atlanta, Ga.

Ranck, of Philadelphia, amused and entertained the ladies with his exhibition of rattlesnakes.

Bell, of Brooklyn, forgot all about the crops in Virginia about election time, and is a thorough believer in passing the offices around.

One of the chief centres of attraction during the Convention was the headquarters of the Army Legislation Committee, presided over by Veterinarian Huidekoper, ably assisted at times by Knowles, of Montana, and Beckett, of Massachusetts.

Conkey, of Grand Rapids, said that while the weather was hot the Convention was a cold place for strangers.

Heyde, of St. Louis, Mo., indicated by his card that he must have a cinch on all the breweries of that city.

Dr. James Robertson, of Chicago, Ill., was there to defend the action of the State Veterinary Examining Board for having granted the registration of nearly 700 veterinarians in the "Prairie State."

Dr. E. C. Porter, of New Castle, Pa., could hardly restrain himself from holding a coroner's inquest on the dogs at Parke, Davis & Co.'s, though they were only under the influence of chloretone.

Jopling, of Owosso, Mich., came down to the Convention loaded with heavy ammunition for big game; the JOURNAL seemed a favorite target for his shots.

Clement, of Baltimore, was the centre of much sympathy over his loss of the State Veterinarianship of "Maryland, my Maryland."

Ackerman, of Brooklyn, hid many of the cares and evidence of age and great responsibilities by the removal of the hirsute adornment of his face.

Stewart, the ever-efficient Secretary, had his resignation written out in his inside pocket, but the members of the Convention would not allow him to unbutton his coat for its presentation.

Butler, of Indianapolis, was full of reminiscences, and had a multitude of experiences to relate of civil practice, agricultural college work, live-stock editorship and as a Southern breeder of a cross between razorbacks and Western lard makers.

Dr. D. King Smith, of Toronto, Ont., was a welcome visitor, and added much interest to the meeting by his exhibition of frozen specimens from the college museum.

The Southern press have been quick to recognize with approval the recognition accorded in official place of Southern veterinarians, and view with much interest the growing strength of the veterinary profession in the South.

The JOURNAL is in receipt of many favorable expressions for Atlantic City in 1901.

Some of the ladies who were present at Detroit are mourning the fact that they have no vote at this time. Perhaps the winning influences of the gentler sex upon those of the masculine type will find expression through them and their wishes be granted for Atlantic City.

One enthusiastic member in favor of Atlantic City has written one of the ladies present at Detroit in favor of Atlantic City, on the score that the "boardwalk" is so much better for walking than the streets of Detroit.

Atlantic City will open wide her doors for the veterinarians in September, 1901. Many of the leading hotels have large assembly rooms where the sessions of the Convention could be held.

Charleston, S. C., is active in the field for the place of meeting for 1901, and strongly urges the merits of another visit to the South and the selection of Charleston, with the attractions of its exposition, as a suitable place for 1901. Ex-State Secretary Dr. McInnes, always an active veterinarian and much interested in the advance of the profession in the South, and especially in the Carolinas, is earnestly at work to obtain this recognition for 1901. The pleasure of an ocean coast trip to those in the East is one of the many attractions he urges in behalf of Charleston.

### *Resolutions adopted at Detroit.*

*Whereas,* This our first meeting in this great Convention City of Detroit has been marked by an attendance in excess in number of any prior meeting; and

*Whereas,* The excellent work done by the local committee of arrangements, the cordial reception of the chief magistrate of the city, the courtesies of the press, the intense interest of the Michigan State Association members, the kindness of the ladies of Detroit and vicinity, have all contributed much to the pleasure and profit of our meeting; therefore be it

*Resolved,* That this Association tender their sincere thanks and express their appreciation of the courtesies extended us, and trust that our meeting in this city may contribute much to the welfare and advancement of the profession in Michigan, Canada, and the Central West.

*Whereas,* Cities are now recognizing the importance of dairy and meat-inspection as essential to the protection of public health,

*Be it resolved,* That we commend to their consideration the selection of men specially trained for the execution of their work, in order that it may be effective. The veterinarian, by his training and his knowledge of animals in health and disease, is pre-eminently better qualified for these positions than any other class of people. We would also commend the adoption of the rules and regulations of the Bureau of Animal Industry pertaining to meat-inspection as far as they are applicable to the conditions.

*Whereas,* The bill for the establishment of an organized Veterinary Corps in the United States Army, which passed the U. S. Senate as "Section 14 of S. 4300," a bill for the efficiency of the army (known as the Army Reorganization Bill), and is now under consideration by the Military Committee of the House of Representatives, provides for such a service as has been found necessary and exists in the armies of all civilized countries; and

*Whereas,* Under the system of administration in the U. S. Army an efficient and economical service cannot be had in any other way than that provided for in our bill, any more than any business house could employ thirty or fifty workmen effectively without a foreman or superintendent; and

*Whereas,* Any compromise simply of rank without organization will not increase the efficiency of the army, and will set back the proper recognition of the profession a quarter of a century; and

*Whereas,* The eight thousand veterinarians in the United States to-day represent a profession, and are not to be classed on the pay-list with teamsters, as part of them are in the U. S. Army under the present system,

*Therefore,* We demand an organized Veterinary Corps as provided for in S. 4300 without change or amendment when this bill passes, or as a part of the first Army Bill which does pass in its place when Congress reconvenes, and we will as a body and as individuals use our best and strongest efforts to secure the passage of said bill.

*Whereas,* The ability of the veterinarian to promote the health of the public and of the animals intrusted to his care can be increased only by his more thorough education; therefore be it

*Resolved,* That we believe that those intrusted with the management of the veterinary schools of America should put forth every possible effort to lengthen the period of instruction required for graduation, to strengthen and make uniform the curriculum and to elevate the standard of matriculation examinations.

*Whereas,* We believe that the time has arrived in the history of our profession and in the history of our stock-breeding industry when the experiment stations of our various States and Territories should undertake the work of research in regard to the physiology and diseases of domestic animals in a more comprehensive manner than has yet been done; therefore be it

*Resolved,* That this Association recommend to the governing boards of the various stations that they give this branch of station work more liberal support.

*Whereas,* The American Veterinary Medical Association has learned with much satisfaction and pleasure of the recent amalgamation of the three State Societies of New Jersey into the Veterinary Medical Association of New Jersey; and,

*Whereas,* In place of factional discord and weakness, we have, as the result of this amalgamation, unity, harmony, and strength in the profession throughout that State; therefore, be it

*Resolved,* That the congratulations of this Association be extended to the profession of the State of New Jersey and to that indefatigable worker and organizer, Dr. Wm. H. Lowe, of this Association, through whose labors this much to be desired event has been accomplished.

*Whereas,* It has pleased Almighty God to remove from our midst our esteemed colleague, Dr. Maurice O. Connell, of Massachusetts; therefore, be it

*Resolved,* That this Association expresses its sincere regrets and extends to his family its sincere sympathy in their great loss; and be it further

*Resolved,* That these resolutions be spread on the minutes and a copy sent to the bereaved family.

*Whereas,* It has pleased Almighty God to remove from our midst our esteemed colleague, Dr. C. A. Mackey, of New York; therefore, be it

*Resolved,* That this Association expresses its sincere regrets and extends to his family its sincere sympathy in their great loss; and be it further

*Resolved,* That these resolutions be spread upon the minutes and a copy sent to the bereaved family.

*Whereas,* It has pleased Almighty God to remove from our midst our esteemed colleague, Dr. E. M. Heath, of Connecticut; therefore, be it

*Resolved,* That this Association expresses its sincere regrets and extends to his family its sincere sympathy in their great loss; and be it further

*Resolved,* That these resolutions be spread upon the minutes and a copy sent to the bereaved family.

*Whereas,* It has pleased Almighty God to remove from our midst our esteemed colleague, Dr. M. J. Tewey, of New York; therefore, be it

*Resolved,* That this Association expresses its sincere regrets, and extends to his family its sincere sympathy in their great loss; and be it further

*Resolved,* That these resolutions be spread upon the minutes and a copy sent to the bereaved family.

W. Horace Hoskins,
Austin Peters,
Wm. Herbert Lowe,
A. W. Bitting,
John J. Repp,
Committee.

